# Determinants of acceptance and usage of generative AI among Chinese medical students: a UTAUT-based empirical investigation

**DOI:** 10.3389/fpsyg.2026.1744827

**Published:** 2026-02-17

**Authors:** Xue Jiang, Weifeng Tong, Mingquan Xue, Zitong Yuan, Jing Tong, Dawei Xu, Haiyang Li

**Affiliations:** 1 School of Stomatology, Xuzhou Medical University, Xuzhou, China; 2 School of Public Health, Xuzhou Medical University, Xuzhou, China; 3 China University of Mining and Technology, Xuzhou, China; 4 Department of Student Affairs, Xuzhou Medical University, Xuzhou, China; 5 Shenyang Heping District Disease Prevention and Control Centers, Shenyang, China; 6 Institute of Medical Humanities, Xuzhou Medical University, Xuzhou, China

**Keywords:** Chinese medical students, generative artificial intelligence (GenAI), higher education, medical education, technology acceptance, UTAUT

## Abstract

**Background:**

Generative artificial intelligence (GenAI) is rapidly transforming higher education, yet empirical evidence remains limited on the factors associated with its acceptance and usage among medical students, especially in non-Western, high-stakes educational contexts such as China. A clear and contextualized understanding of these mechanism is essential to effectively integrate GenAI into medical curricula and prepare future healthcare professionals for AI-augmented clinical practice. Grounded in the Unified Theory of Acceptance and Use of Technology (UTAUT) framework, this study systematically investigated the relationships between core UTAUT constructs, and Chinese medical students’ behavioral intention (BI) and actual usage (AU) of GenAI, testing direct, mediating, and exploratory moderated pathways.

**Methods:**

A cross-sectional online survey was administered to students at a public medical university in China from October 2024 to January 2025, yielding 1781 valid responses. Validated scales were used to measure core UTAUT constructs: performance expectancy (PE), effort expectancy (EE), social influence (SI), facilitating conditions (FCs), BI, and AU. Partial Least Squares Structural Equation Modeling (PLS-SEM) was employed to test the hypothesized relationships.

**Results:**

The model demonstrated strong explanatory power, accounting for 67.6% of the variance in BI and 66.3% in AU. PE (*β* = 0.377, *p* < 0.001), FCs (*β* = 0.333, *p* < 0.001) and SI (*β* = 0.212, *p* < 0.001) were positively associated with BI. EE showed no significant direct association with BI (*β* = 0.038, *p* = 0.209) but had a weak yet significant direct association with AU (*β* = 0.057, *p* = 0.045). BI served as a significant mediator in the relationships between PE, SI, FCs, and AU (all *p* < 0.001) but failed to mediate the association between EE and AU (*p* = 0.219). Age was the only significant moderator for the path from EE to BI (*β* = 0.071, *p* = 0.043) and the path from BI to AU (*β* = 0.024, *p* = 0.022); gender, major, and academic level showed no moderating effects.

**Conclusion:**

This study empirically validates and extends the UTAUT framework within Chinese medical education. Key findings underscore the important roles of PE, FCs and SI, reveal the context-dependent role of EE, and identify the moderating effect of age. Strategic interventions including demonstrating GenAI’s tangible utility, improving technical infrastructure, leveraging peer / faculty advocacy, and tailing strategies to age-related differences are recommended. These insights provide evidence-based guidance for educators, policymakers, and AI developers to support responsible integration of GenAI into medical education, ultimately preparing future healthcare professionals for an AI-driven healthcare ecosystem.

## 1 Introduction

Generative artificial intelligence (GenAI), exemplified by platforms such as ChatGPT and DeepSeek, has emerged as a transformative force in global higher education (Chris Stokel-Walker, 2023; Velli and Zafiropoulos, 2024), fundamentally reshaping pedagogical paradigms and learning behaviors (Harari, 2017; Velli and Zafiropoulos, 2024). By generating human-like content, GenAI offers unprecedented potential to enhance academic efficiency, enable personalized learning and provide professional support (Xie et al., 2024; Zhu et al., 2025). Its adoption is growing, with almost one-third university students already using it for tasks (Wang et al., 2023; Xu et al., 2025), such as brainstorming (Dogru et al., 2024; Rojas, 2024), scientific writing (AlAfnan et al., 2023)^,^ and multilingual translation (Arango-Ibanez et al., 2024; Mao et al., 2024). Beyond direct academic applications, GenAI also contributes to students’ mental well-being (Tam et al., 2023), career preparation (Ivanov and Soliman, 2023), and teaching dynamics (Fatima et al., 2024; Naqa et al., 2023).

While GenAI’s disruptive potential is widely acknowledged, empirical research on its acceptance and adoption remains disproportionately focused on general student populations or non-specialized disciplines (Chiu, 2024; Dogru et al., 2024; Duan et al., 2025). This leaves a critical gap in understanding how GenAI integrates into high-stakes, specialized fields such as medical education, particularly within distinct sociocultural contexts like China (Tao et al., 2024). Given that medical students are future frontline healthcare providers, a context-specific understanding of factors associated with their acceptance and adoption is essential. Such understanding will support the effective integration of this technology into medical training and help prepare a workforce adept in AI-augmented clinical practice (Ghorashi et al., 2023).

### 1.1 GenAI in medical education: the unique context of Chinese medical students

Medical students represent a crucial group for GenAI adoption, often holding complex and ambivalent attitudes that balance enthusiasm for its potential benefits with concerns regarding its reliability and implications (Li and Qin, 2023; Pusic et al., 2024). This is especially pronounced in China, where medical education operates within a unique ecosystem characterized by intense academic pressure, collectivist cultural norm, and an extended, competency-based training pathway to qualification (Duan et al., 2025; Tao et al., 2024). Students must master vast amounts of complex specialized knowledge while succeeding in high-stakes examinations and clinical evaluations throughout nearly a decade of combined undergraduate, postgraduate, and standardized residency training to obtain physician certification (Tao et al., 2024).

Within this demanding environment, GenAI-powered tools, such as intelligent tutoring systems and virtual patient simulators (Geraci et al., 2024; Narayanan et al., 2023; Tang et al., 2018; Tolsgaard et al., 2023), hold substantial promise. They can assist medical students in managing academic workloads, support the development of clinical decision-making skills, facilitate personalized learning and help bridge the gap between theoretical knowledge and clinical practice (Ghorashi et al., 2023; Narayanan et al., 2023; Sunmboye et al., 2025; Wu et al., 2024). However, realizing these potential hinges on a critical precondition: Chinese medical students’ acceptance and usage of GenAI. Identifying the factors and mechanisms underlying their adoption intentions and subsequent behaviors, an area that remains underexplored, is therefore a critical and timely research imperative.

### 1.2 Theoretical framework

To systematically examine related factors of GenAI acceptance and adoption, this study employs the Unified Theory of Acceptance and Use of Technology (UTAUT). UTAUT integrates core constructs form eight foundational technology acceptance models into a comprehensive theoretical framework. It posits that behavioral intention (BI) and actual usage (AU) are influenced by four key antecedents: performance expectancy (PE), effort expectancy (EE), social influence (SI), and facilitating conditions (FCs) (Venkatesh, 2022; Venkatesh et al., 2003). The framework also proposes that sociodemographic factors (e.g., age, gender) may moderate these relationships (Ma et al., 2025; Strzelecki, 2024).

UTAUT has demonstrated robust explanatory power across diverse educational technology contexts, including virtual learning (Efiloğlu Kurt and Tingöy, 2017; Noble et al., 2022), mobile learning (Hoi, 2020), and emerging AI applications such as Chatbot (Tian et al., 2024). Its integrative nature and established validity in predicting adoption within structured environments make it particularly suitable for examining technology acceptance in the rigorous context of medical education (Venkatesh et al., 2003; Xu et al., 2025). Although a growing number of studies have applied UTAUT to understand GenAI adoption in general higher education (Ma et al., 2025; Yilmaz et al., 2024; Zou and Huang, 2023), its application to the distinct, high-stakes Chinese medical educational context remains nascent, presenting a valuable opportunity for contextualized theory testing and extension.

### 1.3 Research gaps and the present study

Against this theoretical and contextual backdrop, three main research gaps motivate the present study. First, existing GenAI acceptance studies primarily focus on general university populations (Sabraz Nawaz et al., 2024; Xu et al., 2025; Zhu et al., 2025), or specific task-based applications (e.g., academic writing and literature reading) (Pan et al., 2025; Zou and Huang, 2023), leaving the unique group of Chinese medical students critically underexplored. Second, prior findings regarding certain UTAUT constructs, (e.g., the relationships between EE, SI and BI) have mixed across different settings (Foroughi et al., 2024; Sobaih et al., 2024). These inconsistencies highlight the need for context-specific investigation into clarify how these relationships manifest within the unique socio-educational ecosystem of Chinese medical education, where the strength of UTAUT pathways may be refigured. Third, although UTAUT acknowledges that individual differences may moderate technology adoption pathways (Venkatesh, 2022; Venkatesh et al., 2003), the moderating roles of sociodemographic variables remain unclear among Chinese medical students. For instance, while Ma et al. found that gender, region and educational background moderate the relationship between BI and AU of GenAI among Chinese users (Ma et al., 2025), Strzelecki (2024) reported non-significant moderating effects of gender. Thus, the potential effects of variables such as age, gender, academic level and major warrant exploratory examination within this population.

To address these gaps, this study aims to: (1) examine the direct associations between core UTAUT constructs (PE, EE, SI, FCs), and BI and AU of GenAI among Chinese medical students; (2) assess the mediating role of BI in linking UTAUT antecedents to AU; and (3) explore the moderating effects of sociodemographic variables, including age, gender, academic level and major, on the hypothesized relationships.

### 1.4 Hypotheses development and conceptual model

**Figure 1:**
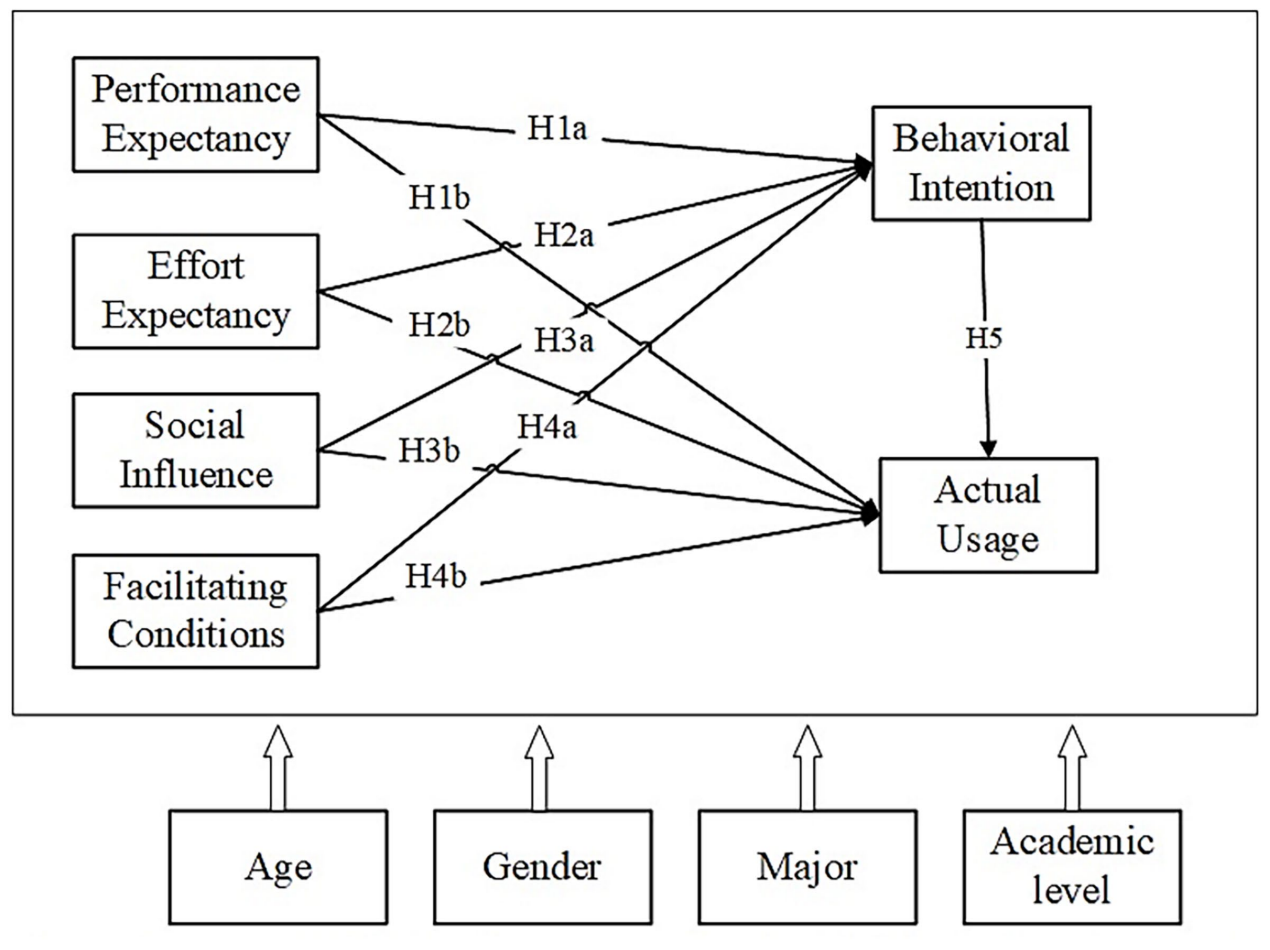
Conceptual model of factors associated with GenAI acceptance and usage among Chinese medical students.

Drawing on UTAUT framework and extant literature, this study develops following hypotheses and proposes a conceptual model for Chinese medical students in the context of GenAI (Figure 1). The model depicts the hypothesized direct and mediating pathways among study constructs, and exploratory moderating effects of sociodemographic variables.

#### 1.4.1 Hypothesized direct relationships

PE refers to the perceived usefulness of using GenAI in enhancing academic performance (Venkatesh et al., 2003), which is consistently identified as a dominant predictor of BI across technology acceptance studies (Kumar and Bervell, 2019; Menon and Shilpa, 2023; Zacharis and Nikolopoulou, 2022). For medical students navigating vast curricula and high-stakes exams, the perception that GenAI is instrumental in improving their learning efficiency and outcomes is expected to directly motivate both their adoption intention and subsequent actual engagement with the technology (Ma and Huo, 2023; Rojas, 2024). Accordingly, we propose:

*H1a*: PE is positively associated with BI to use GenAI.

*H1b*: PE is positively associated with AU of GenAI.

EE reflects the perceived ease of using GenAI (Venkatesh, 2022). Consistent findings are yielded: Duong et al. (2023a) demonstrated a positive EE-BI link, while Romero-Rodríguez et al. (2023) observed no significant associations. While ease of use is generally understood lower the initial adoption barriers (Venkatesh, 2022), its role may be more nuanced in performance-driven environments like medical education. For Chinese medical students, a performance-oriented focus may potentially moderate EE’s effect. However, given the increasing intuitiveness of modern GenAI interfaces (Hasanein and Sobaih, 2023), we posit that EE may still related to usage behavior. We thus hypothesize:

*H2a*: EE is positively associated with BI to use GenAI.

*H2b*: EE is positively associated with AU of GenAI.

SI denotes the impact of beliefs from important referents on students’ decisions to use GenAI (Kelly et al., 2023). Social norms and trusted referents’ opinions play a salient role in shaping BI (Sabraz Nawaz et al., 2024; Strzelecki, 2024). Positive endorsements from these referents reinforce the intentions to adopt GenAI and promote actual behaviors (Al-Emran et al., 2024; Hasanein and Sobaih, 2023; Menon and Shilpa, 2023). This may be particular relevant in collective cultural settings like China, where students prioritize peer recommendations and faculty guidance. Thus, we hypothesize:

*H3a*: SI is positively associated with BI to use GenAI.

*H3b*: SI is positively associated with AU of GenAI.

FCs encompass the perceived availability of technical and organizational support necessary for using GenAI (Menon and Shilpa, 2023; Oye et al., 2014). As a consistent direct influencing factor of both BI and AU (Faqih and Jaradat, 2021; Osei et al., 2022; Venkatesh et al., 2012), FCs are expected to be particularly important in resource-intensive medical education, strengthening both the intention to use GenAI and the ability to sustain its usage (Al-Emran et al., 2024; Menon and Shilpa, 2023). We thus propose:

*H4a*: FCs are positively associated with BI to use GenAI.

*H4b*: FCs are positively associated with AU of GenAI.

BI represents students’ willingness and tendency to use GenAI, theorized as a proximal determinant of GenAI use (Al-Emran et al., 2024; Duong et al., 2023b); while AU denotes the frequency and extent of usage after forming BI (Venkatesh et al., 2003). For Chinese medical students, a strong BI reflects a deliberate commitment to integrating GenAI into learning routines, making it likely translate into frequent and purposeful usage ^55^. Therefore, we posit:

*H5*: BI is positively associated with AU of GenAI.

#### 1.4.2 Hypothesized mediating relationships

UTAUT posits BI as the primary mediating mechanism that links individual perceptions (PE, EE, SI, FCs) to actual behaviors (AU) (Hoi, 2020; Venkatesh et al., 2003; Venkatesh et al., 2012). We expect this proposition to hold for GenAI adoption among Chinese medical students.

*H6*: BI mediates the relationship between PE and AU.

*H7*: BI mediates the relationship between EE and AU.

*H8*: BI mediates the relationship between FCs and AU.

*H9*: BI mediates the relationship between SI and AU.

## 2 Methods

### 2.1 Survey design and data collection

This cross-sectional online survey was administered to students from Xuzhou Medical University, a public medical institution in China. A simple random sampling approach was used to recruit participants from specialties allied to medicine within the university.

Data was collected via the Wenjuanxing (a professional online survey platform widely used in academic research in China) from October 10, 2024 to January 31, 2025. Invitations were disseminated through WeChat and other web-based channels to maximize participant reach and engagement. The study received ethical approval from the Xuzhou Medical University’s Institutional Review Board (IRB Approval No: XMUs-24069) and complied rigorously with the principles of the Declaration of Helsinki. All participants were fully informed of the study’s purposes and electrical informed consent was obtained prior to the survey. Voluntary participation, anonymity, and the right to withdraw at any stage were guaranteed.

### 2.2 Sample size

Sample size was determined based on Nunnally’s criterion (a 10:1 subject-to-item ratio), a widely accepted standard for scale validation studies (Ajzen and Fishbein, 1972). The final survey instrument contained 26 items, resulting in a minimum required sample size of 260.

To ensure data quality, responses completed in less than 3 min or displaying repetitive answering patterns were excluded (Rafique et al., 2020; Sobaih et al., 2024). The online survey platform enforced mandatory response fields, yielding a complete dataset with no missing values. The final sample included 1781 valid responses, substantially exceeding the minimum requirement and ensuring adequate statistical power for subsequent analyses (Streiner, 2003).

### 2.3 Measurement instruments

The survey instrument was developed based on the UTAUT framework and validated scales from extant literature (Duan et al., 2025; Sobaih et al., 2024; Xu et al., 2025). It was structured into three sections:

1. Ethics and Informed Consent: Outlined the study’s purposes, emphasized restricted research-only use, and clarified participants’ rights;
2. Sociodemographic Characteristics: Included age, gender, academic level and major as potential moderators (Taherdoost, 2018);
3. UTAUT constructs (PE, EE, SI, FCs, BI and AU) were measured using adapted and validated scales from prior technology acceptance studies (Alabdullah et al., 2020; Kijsanayotin et al., 2009; Li and Qin, 2023; Sobaih et al., 2024). All items for PE, EE, SI, FCs, and BI were measured on 7-point Likert scales ranging from “strongly disagree” to “strongly agree.” AU was assessed using a 5-point frequency scale (“never” to “very frequently”) to self-report the usage behavior more accurately. Higher scores indicate stronger agreement or more frequent usage. Item wording was refined and revised based on feedback from medical students and scholars to enhance contextual relevance and content validity (Nunally, 1978; Tian et al., 2024).

### 2.4 Statistical analysis

Descriptive analyses were conducted using SPSS 25.0. The data were further examined using Partial Least Squares Structural Equation Modeling (PLS-SEM) via Smart PLS 4.0. PLS-SEM was chosen for its robustness with non-normal data, flexibility in sample size requirements, and suitability for predictive modeling of complex hypothesized framework (Hair et al., 2017b; Hair et al., 2012; Ringle et al., 2020; Suliman et al., 2024). This methodological approach is well-established in UTAUT-based research (Rahman et al., 2021; Rangel and Humphrey-Murto, 2024). For all statistical analyses, *α* = 0.05 was applied.

The analysis was conducted following two phases (Cabero-Almenara et al., 2024; Hair et al., 2017b): measurement model evaluation and structural model testing.

### 2.5 Measurement model assessment

The measurement model was assessed for internal consistency, convergent validity, and discriminant validity: Internal consistency was evaluated using Cronbach’s α and composite reliability (CR), with values above 0.70 considered acceptable (Fornell and Larcker, 1981; Hair et al., 2017b). Convergent validity was established if the average variance extracted (AVE) exceeded 0.50 and factor loadings surpassed 0.70 (Carmines and Zeller, 1979; Fornell and Larcker, 1981). Discriminant validity was evaluated using the Fornell-Larcker criterion and heterotrait-monotrait (HTMT) ratio, with a standard threshold of 0.90 used to indicate adequate discrimination (Fornell and Larcker, 1981; Henseler et al., 2015). Additionally, collinearity was assessed via using Variance Inflation Factors (VIF), with values below 10 indicating no significant multicollinearity (O’brien, 2007; Rahman et al., 2021).

### 2.6 Structural model assessment

The hypothesized relationship (direct, mediating, and moderating pathways) were tested using the structural model. Path coefficients and their significance were estimated via a bootstrapping procedure with 5,000 resamples (Preacher and Hayes, 2008). A path was considered significant if its 95% confidence interval (CI) did not include 0. The explanatory power of the structural model was evaluated using R^2^ values. Model fit was assessed using the Standardized Root Mean Square Residual (SRMR) and the Normed Fit Index (NFI) (Henseler and Sarstedt, 2013).

## 3 Results

### 3.1 Descriptive characteristics

Table 1 summarized the sociodemographic characteristics of the 1781 participants. Most of the participants (98.8%) were 25 years old or younger. Female participants (1,113, 62.5%) outnumbered male participants (668, 37.5%). Undergraduate students constituted the overwhelming majority (98.1%). The sample was diverse in terms of majors, with the highest representation from Stomatology (26.3%), Clinical Medicine (19.1%), and other medical specialties (15.4%).

**Table 1:** Sociodemographic characteristics of the participants (*N* = 1781).

Variables	Items	Frequency (%)
Age	Lower than 20 years	1,180 (66.3%)
	21–25 years	578 (32.5%)
	26–30 years	11 (0.6%)
	31–35 years	3 (0.2%)
	More than 35 years	9 (0.5%)
Gender	Male	668 (37.5%)
	Female	1,113 (62.5%)
Academic level	Undergraduate students	1748 (98.1%)
	Master postgraduate students	23 (1.3%)
	Doctoral students	10 (0.6%)
Major	Clinical medicine	340 (19.1%)
	Anesthesiology	197 (11.1%)
	Stomatology	468 (26.3%)
	Nursing	167 (9.4%)
	Medical imaging	113 (6.3%)
	Laboratory medicine	36 (2.0%)
	Other medical specialties	275 (15.4%)
	Other medical-related disciplines	185 (10.4%)

Table 2 presented the descriptive statistics for core UTAUT constructs. PE had the highest mean score (5.913 ± 1.069), followed by BI (5.340 ± 1.150), while self-reported AU had the lowest mean (3.952 ± 0.754).

**Table 2:** Descriptive statistics of core UTAUT constructs (*N* = 1781).

Variables	Minimum	Maximum	Mean	Standard deviation
BI	1	7	5.340	1.150
PE	1	7	5.913	1.069
EE	1	7	5.083	1.250
SI	1	7	5.074	1.351
FCs	1	7	5.040	1.270
AU	1	5	3.952	0.754

BI, Behavioral Intention; PE, Performance Expectancy; EE, Effort Expectancy; SI, Social Influence; FC, Facilitating Conditions; AU, Actual Usage.

### 3.2 Measurement model validation

As summarized in Table 3–5, the measurement model demonstrated robust psychometric properties. All constructs showed high internal consistency: Cronbach’s α values ranged from 0.902 to 0.965, and CR values ranged from 0.939 to 0.975, each exceeding the conventional threshold of 0.70. Convergent validity was established, with all AVE values falling between 0.836 and 0.917, well above the recommended 0.50 benchmark. All standardized factor loadings ranged from 0.897 to 0.963, further confirming strong item-convergence (Fornell and Larcker, 1981). Discriminant validity was established using the Fornell –Larcker criterion (Table 4), with the square root of AVE for each construct (range: 0.915–0.958), exceeding its correlations with all other constructs (0.504–0.769) (Fornell and Larcker, 1981). Additionally, all HTMT values (range: 0.524–0.827) were below 0.90, confirming the conceptual distinctiveness of the constructs (Table 5). Finally, VIF values for all constructs were below 5 (range: 1.684–3.098; Table 6), indicating no multicollinearity concerns (Kock and Lynn, 2012; O’brien, 2007).

**Table 3:** Factor loadings, composite reliability and average variance for each construct.

Construct	Item	Factor loading	Cronbach’s α	CR	AVE
AU	AU1	0.946	0.945	0.965	0.901
	AU2	0.953			
	AU3	0.948			
BI	BI1	0.923	0.914	0.946	0.853
	BI2	0.950			
	BI3	0.897			
EE	EE1	0.905	0.902	0.939	0.836
	EE2	0.905			
	EE3	0.933			
FCs	FC1	0.934	0.953	0.966	0.877
	FC2	0.949			
	FC3	0.953			
	FC4	0.909			
PE	PE1	0.956	0.965	0.975	0.906
	PE2	0.947			
	PE3	0.96			
	PE4	0.945			
SI	SI1	0.954	0.955	0.971	0.917
	SI2	0.963			
	SI3	0.955			

CR, Composite Reliability; AVE, Average Variance Extracted; AU, Actual Usage; BI, Behavioral Intention; EE, Effort Expectancy; FCs, Facilitating Conditions; PE, Performance Expectancy; SI, Social Influence.

**Table 4:** Discriminant validity based on the Fornell and Larcker method.

Variables	AU	BI	EE	FCs	PE	SI
AU	0.949					
BI	0.769	0.924				
EE	0.623	0.639	0.915			
FCs	0.661	0.709	0.735	0.937		
PE	0.668	0.688	0.548	0.504	0.952	
SI	0.655	0.684	0.685	0.675	0.567	0.958

AU, Actual Usage; BI, Behavioral Intention; EE, Effort Expectancy; FCs, Facilitating Conditions; PE, Performance Expectancy; SI, Social Influence.

**Table 5:** Heterotrait-monotrait ratio (HTMT) values.

Variables	AU	BI	EE	FCs	PE	SI
AU						
BI	0.827					
EE	0.672	0.701				
FCs	0.696	0.759	0.791			
PE	0.699	0.730	0.583	0.524		
SI	0.689	0.732	0.735	0.706	0.590	

AU, Actual Usage; BI, Behavioral Intention; EE, Effort Expectancy; FCs, Facilitating Conditions; PE, Performance Expectancy; SI, Social Influence.

**Table 6:** Results of the variables’ collinearity indicators.

Paths	VIF	f^2^
BI - > AU	3.098	0.147
EE - > AU	2.670	0.004
EE - > BI	2.924	0.002
FCs - > AU	2.898	0.021
FCs - > BI	2.788	0.124
PE - > AU	2.095	0.071
PE - > BI	1.684	0.265
SI - > AU	2.461	0.018
SI - > BI	2.402	0.059

AU, Actual Usage; BI, Behavioral Intention; EE, Effort Expectancy; FCs, Facilitating Conditions; PE, Performance Expectancy; SI, Social Influence.

### 3.3 Structural model and hypotheses testing

#### 3.3.1 Direct associations

**Figure 2:**
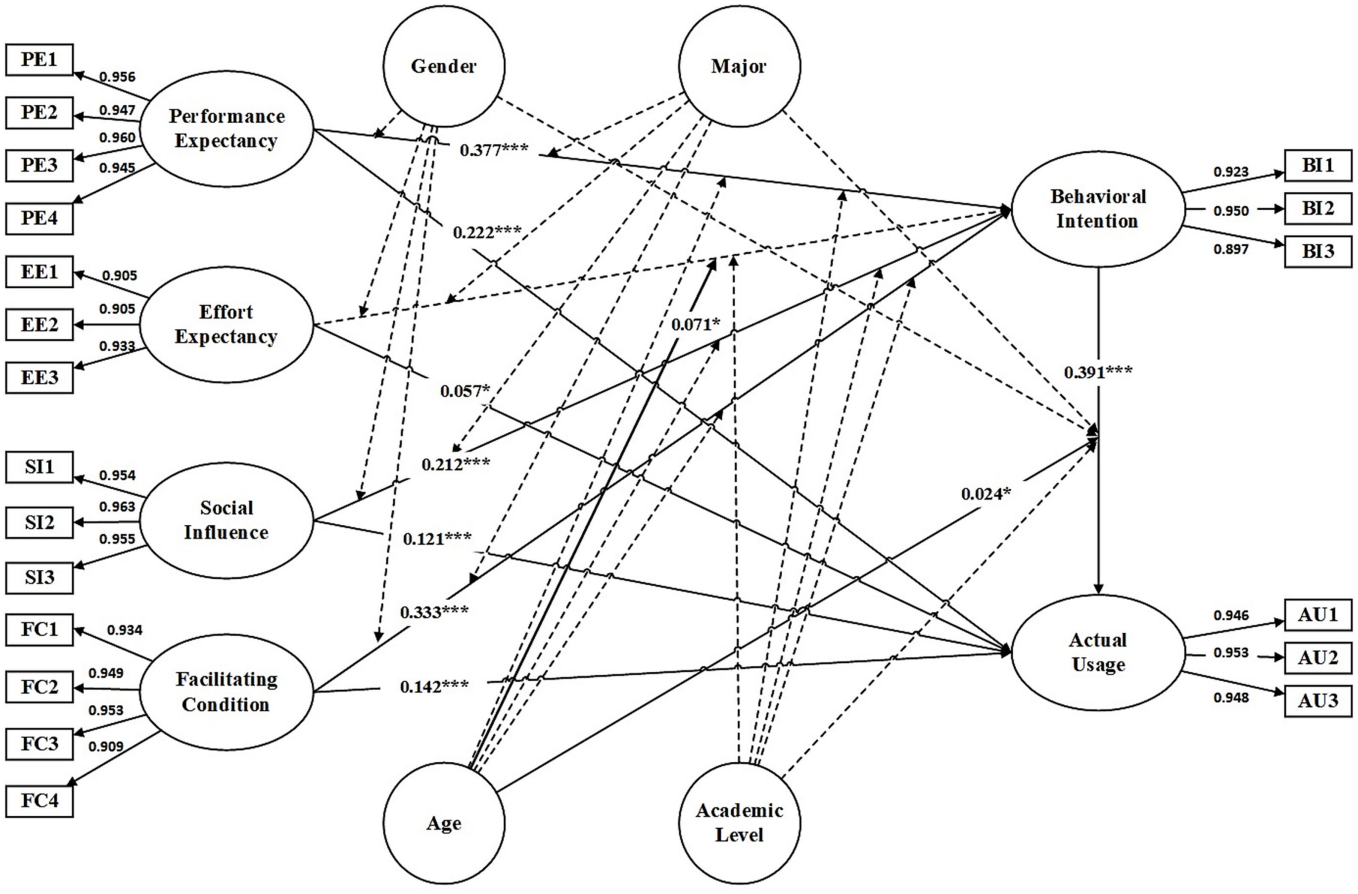
The structural model with path conditions. *p < 0.05, **p < 0.01, ***p < 0.001.

Table 7 presented the results of hypothesized direct relationships, including standardized path coefficients and their significance levels, while Figure 2 illustrated the structural model with corresponding paths.

**Table 7:** Path coefficients and results of the hypotheses tests.

Hypothesis	Direct paths	*β*	*T*-value	*p*-value	Decision
H5	BI - > AU	0.391	11.206	<0.001	Accepted
H2b	EE - > AU	0.057	2.002	0.045	Accepted
H2a	EE - > BI	0.038	1.255	0.209	Rejected
H4b	FCs - > AU	0.142	4.536	<0.001	Accepted
H4a	FCs - > BI	0.333	9.734	<0.001	Accepted
H1b	PE - > AU	0.222	8.373	<0.001	Accepted
H1a	PE - > BI	0.377	16.74	<0.001	Accepted
H3b	SI - > AU	0.121	3.954	<0.001	Accepted
H3a	SI - > BI	0.212	7.237	<0.001	Accepted

AU, Actual Usage; BI, Behavioral Intention; EE, Effort Expectancy; FCs, Facilitating Conditions; PE, Performance Expectancy; SI, Social Influence.

PE (*β* = 0.377 *p* < 0.001), FCs (*β* = 0.333, *p* < 0.001), and SI (*β* = 0.212, *p* < 0.001) demonstrated significant positive associations with BI, supporting H1a, H3a and H4a, respectively. EE (*β* = 0.038, *p* = 0.209) was not significantly associated with BI, leading to the rejection of H2a.

BI exhibited a strong and positive association with AU (*β* = 0.391, *p* < 0.001), supporting H5. PE (*β* = 0.222, *p* < 0.001), FCs (*β* = 0.142, *p* < 0.001), SI (*β* = 0.121, *p* < 0.001), and EE (*β* = 0.057, *p* = 0.045) were all significantly and positively associated with AU, supporting H1b, H2b, H3b, H4b, respectively.

#### 3.3.2 Mediation analyses

As detailed in Table 8, BI significantly mediated the associations between PE and AU (*β* = 0.147, *p* < 0.001), SI and AU (*β* = 0.083, *p* < 0.001), and FCs and AU (*β* = 0.132, *p* < 0.001), supporting H6, H8, and H9. The mediating effect of BI between EE and AU was not significant (*β* = 0.015, *p* = 0.219), leading to the rejection of H7.

**Table 8:** Mediating effects and results of the hypotheses tests.

Hypotheses	Paths	*β*	*T*-value	*P*-value	Mediating effect	Decision
H7	EE - > BI - > AU	0.015	1.228	0.219	0.012	Rejected
H8	FCs - > BI - > AU	0.132	7.997	<0.001	0.016	Accepted
H6	PE - > BI - > AU	0.147	9.036	<0.001	0.016	Accepted
H9	SI - > BI - > AU	0.083	5.796	<0.001	0.014	Accepted

AU, Actual Usage; BI, Behavioral Intention; EE, Effort Expectancy; FCs, Facilitating Conditions; PE, Performance Expectancy; SI, Social Influence.

#### 3.3.3 Exploratory moderation analyses

The results of exploratory moderation analyses were summarized in Table 9. Gender, major, and academic level showed no significant moderating effects on any of the hypothesized paths (all *p* > 0.05). Only age was found to be a significant moderator in two specific paths: it significantly moderated the relationship between EE and BI (*β* = 0.071, *p* = 0.043), and the relationship between BI and AU (*β* = 0.024, *p* = 0.022). No other moderating effects of age were statistically significant.

**Table 9:** Exploratory moderating effects.

Paths	*β*	*T*-value	*P*-value	Moderating effect
Age × EE - > BI	0.071	2.025	0.043	0.035
Age × PE - > BI	0.013	0.539	0.590	0.025
Age × FC - > BI	−0.063	1.518	0.129	0.042
Age × SI - > BI	−0.01	0.27	0.787	0.036
Age × BI - > AU	0.024	2.298	0.022	0.011
Gender × FC - > BI	0.077	1.283	0.200	0.06
Gender × SI - > BI	−0.078	1.388	0.165	0.056
Gender × EE - > BI	−0.006	0.226	0.821	0.028
Gender × PE - > BI	0.004	0.166	0.868	0.026
Gender × BI - > AU	−0.002	0.285	0.776	0.007
Major × FC - > BI	−0.016	0.44	0.660	0.037
Major × SI - > BI	−0.003	0.117	0.907	0.029
Major × EE - > BI	−0.002	0.05	0.960	0.031
Major × PE - > BI	0.02	0.909	0.364	0.022
Major × BI - > AU	0.026	1.63	0.103	0.016
Degree × FC - > BI	0.077	1.283	0.200	0.06
Degree × SI - > BI	−0.078	1.388	0.165	0.056
Degree × EE - > BI	−0.006	0.226	0.821	0.028
Degree × PE - > BI	0.004	0.166	0.868	0.026
Degree × BI - > AU	−0.002	0.285	0.776	0.007

AU, Actual Usage; BI, Behavioral Intention; EE, Effort Expectancy; FCs, Facilitating Conditions; PE, Performance Expectancy; SI, Social Influence.

### 3.4 Model fit and explanatory power

The structural model demonstrated a satisfactory fit to the data (Table 10): SRMR = 0.029 < 0.080, NFI = 0.932 > 0.900, indicating an acceptable overall fit (Bentler and Bonett, 1980; Henseler and Sarstedt, 2013). The model demonstrated strong explanatory power, accounting for 67.6% of the variance in BI (*R*^2^ = 0.681, adjusted *R*^2^ = 0.676) and 66.3% in AU (*R*^2^ = 0.666, adjusted *R*^2^ = 0.663).

**Table 10:** The predictive power and goodness-of -fit indices of structural model.

Indices	SRMR	NFI
Model fit	0.029	0.932
Predictive power	R^2^	Adjusted-R^2^
AU	0.666	0.663
BI	0.681	0.676

AU, Actual Usage; BI, Behavioral Intention.

## 4 Discussion

Grounded in the UTAUT framework, this study is among the first to systematically investigate associated factors of GenAI acceptance and usage among Chinese medical students, a critical yet underexplored population within a non-Western, high-stakes educational context. The model demonstrated substantial explanatory power, validating the applicability of UTAUT while revealing nuanced, context-specific relationships (e.g., the context-dependent role of EE). These findings refine the theoretical understanding and provide actionable insights for practice.

### 4.1 The dominant role of PE

Confirming H1a and H1b, PE emerged as the strongest correlate of both BI and AU among Chinese medical students. This finding was consistent with prior research in medical education (Li and Qin, 2023), and further corroborated cross-cultural evidence identifying PE as a pivotal factor of GenAI acceptance (Tian et al., 2024; Xu et al., 2025; Zhu et al., 2025), particularly for academic purposes (Sobaih et al., 2024; Strzelecki, 2024; Toh et al., 2023). Within the high-pressure context of Chinese medical education, characterized by a demanding curricula, high-stakes examinations, and intensive clinical training requirements, the instrumental value of GenAI in enhancing learning efficiency, simplifying complex medical concepts, and optimizing exam preparation serves as a primary motivator for its adoption (Li and Qin, 2023; Sobaih et al., 2024). Specifically, students who perceive GenAI as useful are not only more willing to use it but also more likely to translate that intention into actual engagement. For these individuals, GenAI’s perceived utility in improving academic performance outweighs other considerations, such as ease of use (Dogru et al., 2024; Xu et al., 2025). Consequently, interventions aimed at promoting the adoption and integration of GenAI in this setting should prioritize clearly articulating and empirically demonstrating its tangible academic and clinical benefits.

### 4.2 The enabling role of FCs

The findings supported H4a and H4b, revealing that FCs were significantly associated with both BI and AU among Chinese medical students. This aligned with evidence established in prior e-learning, and mobile learning research (Arain et al., 2019; Suliman et al., 2024). The results highlighted that GenAI adoption is not merely an individual willingness but is substantially enabled by institutional support (Venkatesh et al., 2012). Well-developed FCs, such as reliable infrastructure, institutional backing, and structured training, effectively mitigate practical barriers to technology usage (Ain et al., 2016; Azizi et al., 2020). Within the resource-intensive context of medical education, these conditions allow students to engage with GenAI more confidently (Bati et al., 2024; Menon and Shilpa, 2023) and are crucial for translating positive intentions to sustained usage (Al-Emran et al., 2024; Sobaih et al., 2024). For example, stable internet access and targeted training programs can help students navigate integrate GenAI into personalized learning, whereas unequal access to infrastructure or guidance may exacerbate educational disparities (Hashim et al., 2022). Thus, FCs serve as both a practical enabler of GenAI adoption and a potential lever for promoting equity (Sunmboye et al., 2025). Strategic investments in campus-wide GenAI support systems is essential to maximize adoption and ensure that all students, regardless of their background or technical proficiency, can leverage GenAI to enhance their learning. Such an approach is vital for preventing the widening of digital divides within medical education.

### 4.3 The important role of SI

The results supported H3a and H3b, indicating a significant association between SI and both BI and AU. This finding resonated with the UTAUT framework and prior educational technology research (Wu et al., 2022), including studies on ChatGPT (Strzelecki, 2024) and Chatbot (Tian et al., 2024). This relationship holds particular salience in the collectivist cultural context of China, where individuals attach considerable weight to opinions trusted referents (Sabraz Nawaz et al., 2024; Sobaih et al., 2023; Velli and Zafiropoulos, 2024). For Chinese medical students, endorsements from faculty, peers, and institutional authorities serve as powerful social cues. These cues reduce uncertainty regarding the utility and appropriateness of GenAI (Xu et al., 2025), effectively framing it as a legitimate and valuable educational resource (Sobaih et al., 2024; Tao et al., 2024; Xu et al., 2025). Such social validation, in turn, strengthens adoption intentions and encourages subsequent usage behavior (Tao et al., 2024; Zacharis and Nikolopoulou, 2022). Observable adoption by respected others and positive feedback further enhance students’ perceptions of GenAI’s instrumental value (Sobaih et al., 2024; Tao et al., 2024), creating a reinforcing cycle that solidifies BI (Zacharis and Nikolopoulou, 2022). Broader societal and institutional signals, such as supportive policies or positive media coverage, may further amplify this effect (Tao et al., 2024; Xu et al., 2025). Therefore, cultivating positive social norms through advocacy by respected figures and sharing peer success experiences may be a culturally congruent and effective strategy to promote GenAI adoption.

### 4.4 The context-dependent role of EE

The role of EE in GenAI acceptance and adoption among Chinese medical students emerged as more nuanced and context-dependent. While EE exhibited a weak yet significant direct association with self-reported usage (supporting H2b), it was not significantly associated with BI (rejecting H2a). This pattern aligned with prior studies conducted in structured educational environments (Ain et al., 2016; Bayaga and du Plessis, 2023; Prasetyo et al., 2021; Zacharis and Nikolopoulou, 2022), but diverged from other research where EE significantly predicted BI (Tao et al., 2024; Wu et al., 2022).

This discrepancy can be interpreted within the specific context of Chinese medical education. In this high-stakes, performance-driven setting, the primary concern for Chinese medical students appears to be “Will it help me succeed?” (PE), which may overshadow the question “Is it easy to use?” (EE) when forming intentions to adopt GenAI, even if the technology requires a moderate learning effort (Almahri et al., 2020; Sobaih et al., 2024). Moreover, contemporary GenAI tools such as Chatbots, are becoming increasingly intuitive and user-friendly, thereby lowering adoption barriers related to perceived effort (Cabero-Almenara et al., 2024; Tian et al., 2024). Additionally, the study’s sample of young digital natives, who have repeated exposure and growing familiarity with GenAI, may further reduce the salience of ease-of-use considerations (Venkatesh et al., 2003).

Importantly, the persistent direct link between EE and AU suggested that perceived ease of use many facilitating actual engagement without necessarily shaping motivational intent (Sobaih et al., 2024). This does not diminish the theoretical relevance of EE but highlights its context-dependent role: in utility-maximizing and high-stakes environments such as medical education, EE may act more as a facilitator of sustained behavior than as a motivator of intention. This nuanced finding warrants further investigation in other high-stakes professional educational contexts.

### 4.5 The mediating role of BI

Confirming H5, BI was a strong and direct factor of AU, reinforcing its role as the most proximal determinant of technology engagement (Venkatesh, 2022). Furthermore, BI acted as a significant mediator between PE, SI and FCs with AU (supporting H6, H8, H9), which aligned with prior evidence from technology acceptance research (Jo, 2023; Sobaih et al., 2024; Strzelecki, 2024; Xu et al., 2025). This reaffirmed the core “intention-centered” pathway of UTAUT, consistent with the foundational assumptions of well-established theories such as the Technology Acceptance Model (TAM) (Davis, 1989), and Theory of Planned Behavior (TPB) (Ajzen, 1991). The findings indicated that Chinese medical students’ positive perceptions of GenAI, including its perceived utility, social relevance and supporting conditions, are channeled into AU primarily through motivational intention (Wan and Gu, 2024).

The failure of BI to mediate the relationship between EE and AU (rejecting H7) further underscores the distinctive, context-dependent role of EE in this setting. It suggests that whereas BI acts as the central conduit for most antecedents, EE may facilitate actual behavior through more immediate and heuristic pathways. From a practical standpoint, this highlights the need to combine intention-shaping strategies (e.g., highlighting benefits) with direct usability improvements to foster sustained adoption.

### 4.6 The exploratory moderating role of age

Beyond the direct and mediating pathways, the exploratory moderation analyses identified age as the only significant moderator. It strengthened both the EE → BI and BI → AU paths. This finding extended prior research (Menon and Shilpa, 2023), and introduced a more nuanced understanding of age-related differences in GenAI adoption among Chinese medical students.

The moderating effect of age on the EE-BI path suggested that EE’s effect on BI strengthens with age. Older medical students (e.g., final-year students or those in clinical training stages) may adopt a more pragmatic approach, placing greater value on efficiency due to heightened academic and clinical demands. For these students, ease of use becomes a more salient factor in forming usage intentions, even tools requiring moderate effort may be embraced if they streamline learning process (Alowais et al., 2023). Consistent with this, age also strengthened the BI→AU path. Older students demonstrate grater consistency in translating intentions into actual behaviors, likely reflecting a stronger practical need for efficiency-enhancing GenAI tools to cope with demanding training requirements (Sunmboye et al., 2025). In contrast, younger students may engage with GenAI in a more exploratory way, where ease of use plays a less decisive role in intentional adoption.

The absence of significant moderating effects of gender, major and academic level warranted a cautious interpretation. This may be attributable to the relative homogeneity of our sample, drawn from a single institution with a predominantly undergraduate population. The shared high-stakes educational environment may have overridden potential group-specific differences. Additionally, the uneven distribution (e.g., undergraduates constituting 98.1% of the sample) likely limited variability across academic levels, reducing the ability to detect moderating effects. Future studies should employ more balanced samples across academic levels, genders, and majors, and adopt multi-institutional sampling to re-examine these relationships.

### 4.7 Theoretical implications

This study has several theoretical contributions.

Primarily, it validates and contextually extends the UTAUT framework to the underexplored domain of GenAI acceptance and adoption among Chinese medical students. The framework retained strong explanatory power, extending UTAUT’s applicability beyond general educational settings to specialized, high-stakes professional learning environments.

A principal theoretical insight is the context-dependent role of EE. The decoupling of EE from BI, alongside its persistent direct link to AU, challenges the original proposition of UTAUT. This suggests that in performance-driven, high-utility contexts like medical education, the conventional pathway through which ease-of-use influences intention may be attenuated, while its association with actual behavior remains salient. This finding supports a more contingent view of technology acceptance, wherein the prominence of core constructs may be shaped by contextual priorities (e.g., performance outcomes may outweigh ease of use).

Furthermore, the study introduces a developmental perspective through the moderating role of age. The finding that adoption mechanisms (specifically the EE → BI and BI→AU paths) strengthen for older students indicates that GenAI acceptance is not static but rather dynamic, potentially evolving as students advance in their academic training and clinical responsibilities. This underscores the value of incorporating life-course or stage-based variables into technology adoption research, especially within prolonged and intensive professional programs such as medical education.

### 4.8 Practical implications

The findings translate into a multi-pronged strategy for educators, policymakers and AI developers involved in integrating GenAI into medical education.

Given the paramount importance of PE, educators and instructional designers should prioritize clearly demonstrating GenAI’s instrumental value. Curricular integrations and training should explicitly articulate and showcase tangible academic and clinical benefits, such as simplifying complex concepts or optimizing exam preparation.

The critical role of FCs calls for strategic investment in enabling infrastructure and policies. This includes ensuring reliable technical support such as stable campus-wide internet, access to medical-specific GenAI tools, along with structured training programs (e.g., “GenAI for Clinical Reasoning” workshops) (Tao et al., 2024; Zacharis and Nikolopoulou, 2022). Such investments are not merely operational but fundamental to equitable adoption, helping to prevent a GenAI-driven digital divide among students.

To leverage the power of SI, institutions should actively cultivate positive social norms. Faculty endorsement, and the sharing of peer success stories can legitimize GenAI as a valuable learning tool. Institutional policies that create platforms for sharing best practices and facilitating peer-led demonstrations can effectively reduce uncertainty and promote acceptance.

Additionally, the nuanced findings suggest that support strategies should be tailored to different learner profiles. For example, emphasizing time-saving benefits and ease of use of GenAI may resonate particularly with older students who face high efficiency demands. Fostering engagement through exploratory, utility-driven applications that highlight immediate tangible benefits may serve as a more effective entry point for younger students.

### 4.9 Strengths

This study possesses several notable strengths. First, it focuses on Chinese medical students, an important yet understudied population within a distinctive non-Western, high-stakes educational context, filling a significant gap in GenAI acceptance literature. Second, it extends the UTAUT framework to the emerging GenAI context, providing relevant implications for medical education. Third, the identification of age as a meaningful moderator adds a developmental perspective for understanding GenAI adoption. Additionally, the use of a large sample and a rigorous analytic approach (PLS-SEM) enhances the statistical robustness and reliability of the results (Hair et al., 2011).

### 4.10 Limitations and future research

Notwithstanding these strengths, several limitations must be acknowledged to properly contextualize the findings and guide future research.

First, this study established associative relationships but cannot support causal conclusions, as cross-sectional data precludes inferences about temporal order or causality. Future research should employ longitudinal designs that track students from pre-clinical to clinical stages to capture dynamic changes in GenAI adoption over time and to validate the hypothesized UTAUT pathways (Sunmboye et al., 2025). Experimental designs (e.g., randomized controlled trials of GenAI training interventions) could further help establish causal links between key constructs.

Second, reliance on self-reported measures on all constructs (e.g., AU, which is based on self-reported measures rather than objective behavioral data) introduced the potential risk of common method bias and social desirability bias. To address these concerns, procedural remedies (e.g., anonymous data collection, well-validated scales) were employed. Additionally, a common method factor test was conducted to assess the potential common method variance. Using confirmatory factor analyses, the fit of competing models was compared (Podsakoff et al., 2003; Williams et al., 2010). The null model demonstrated poorer model fit (χ^2^/df = 93.555, GFI = 0.448, CFI = 0.633, TLI = 0.590, RMSEA = 0.228) than the measurement–plus-method model (χ^2^/df = 7.347, GFI = 0.937, CFI = 0.977, TLI = 0.972, RMSEA = 0.060). The results indicated that a single method factor did not account for the covariance among the measures, suggesting common method bias was not a serious concern in this study (Podsakoff et al., 2003). Future studies should incorporate objective behavioral data (e.g., GenAI usage logs, platform analytics) and multisource assessments (e.g., faculty ratings of student’s GenAI engagement) to enhance validity (Chan and Zary, 2019; Prakash et al., 2022; Sapci and Sapci, 2020).

Third, data were collected from a single institution, which, while pragmatic (Sunmboye et al., 2025), limited the generalizability of the findings. Future research should adopt stratified sampling across multiple, diverse institutions (e.g., varying in region, type, and resources) to enhance external validity and representativeness.

Fourth, although UTAUT demonstrated strong explanatory power, other related psychological variables such as AI anxiety, perceived risk and personal innovativeness were not included. Integrating these variables would provide a more comprehensive understanding of the mechanisms underlying GenAI adoption among medical students.

Finally, the non-significant moderating effects of gender, major, and academic level should be interpreted cautiously. These findings may be attributable to sample characteristics (e.g., relative homogeneity, imbalanced distribution) or to the potentially overriding influence of the shared educational context. Future studies with more diverse and balanced samples, drawn from institutions, academic stages, and demographic groups, is needed to re-examine these relationships.

## 5 Conclusion

This study is among the first to empirically apply and validate UTAUT framework for understanding GenAI acceptance and adoption among medical students in China. The findings confirm that PE, SI, and FCs are significant enablers of adoption and subsequent usage behaviors of GenAI. A notable contextual insight is the non-significant association between EE and BI, suggesting that ease of use is secondary to perceived utility in this high-stakes learning environment. Furthermore, age was found to moderate EE-BI and BI-AU pathways.

Theoretically, this research extends UTAUT by demonstrating how its core relationships could be reshaped within specific cultural and educational settings. Practically, it offered clear guidance: educators and institutions should demonstrate GenAI’s instrumental value and establish robust support infrastructures, while policymakers and developers need to design context-sensitive implementation strategies.

Overall, this study provides a foundational framework for integrating GenAI into medical education, with the aim of effectively equipping future healthcare professionals for an increasingly AI-augmented clinical landscape.

## Data Availability

The raw data supporting the conclusions of this article will be made available by the authors, without undue reservation.
